# Evaluation of the nutritional and digestive impact of irradiation in nasopharyngeal cancers: the French multicenter retrospective DRAKAR study

**DOI:** 10.1016/j.ctro.2026.101303

**Published:** 2026-10-06

**Authors:** Thomas Cochin, Jennifer Le Guévelou, Juliette Thariat, Justine Lequesne, Sébastien Thureau, Alexandre Coutte, Jacques Balosso, Jonathan Lichtlé, Emmanuel Kammerer, Joël Castelli, Xavier Liem, Sylvie Khan, Audrey Rambeau, Audrey Larnaudie

**Affiliations:** a Department of Radiation Oncology, François Baclesse Center, Caen, France; b Department of Radiation Oncology, Eugène Marquis Center, Rennes, France; c Department of Statistics, François Baclesse Center, Caen, France; d Department of Radiation Oncology, Henri Becquerel Center, QuantIF-AIMS, University of Rouen, Rouen, France; e Department of Radiation Oncology, Amiens University Hospital, Amiens, France; f Department of Radiation Oncology, Grenoble University Hospital, Grenoble, France; g Department of Radiology, Lille University Hospital, Lille, France; h Department of Radiation Oncology, Mallet Proux Center, Laval, France; i Department of Radiotherapy and Brachytherapy, Oscar Lambret Center, Lille, France; j Department of Nutrition, François Baclesse Center, Caen, France; k Department of Medical Oncology, François Baclesse Center, Caen, France

**Keywords:** Nasopharyngeal cancer, Weight loss, Enteral nutrition, Acute radiation toxicities

## Abstract

Objective: Radiotherapy for nasopharyngeal cancers is associated with frequent digestive and nutritional toxicities, weight loss (WL), and malnutrition, criteria of poor tolerance and poor prognosis. The primary objective of DRAKAR was to evaluate the effect of prophylactic enteral nutrition (EN) on weight change (WC) during, at the end of, and after radiotherapy in patients with nasopharyngeal cancer from a non-endemic French population.

Materials and methods: DRAKAR is a retrospective, multicenter study conducted in five French centers. Patients received radiotherapy with curative intent, with or without concurrent chemotherapy, for primary nasopharyngeal cancer between 2015 and 2024. The primary endpoint was WC according to nutritional support modality at the end of radiotherapy. Secondary endpoints included rates of moderate and severe malnutrition (5% or 10% WL in one month for moderate and severe malnutrition), grade ≥ 3 toxicities, dosimetry parameters (volumes, particles), use and duration of EN and parenteral nutrition, prolonged radiotherapy due to treatment interruption, hospitalizations, and overall survival.

Results: Among the 124 patients analyzed (59.7% UCNT, 40.3% squamous cell carcinoma), mean WC was −6.45% at the end of radiotherapy. At one month, 35.5% were malnourished, compared with 8% before treatment. EN was used in 55.6% of patients, including 16.1% prophylactically. Prophylactic EN was associated with stabilization of WC after treatment. Regarding malnutrition, age < 55 years was the only significant clinical factor associated with its occurrence, with no association found with toxicity, dosimetric, or therapeutic characteristics.

Conclusion: In patients treated for nasopharyngeal cancer, prophylactic EN was associated with post-treatment WC stabilization but not with hospitalization or improved survival. Given potential confounding by indication, these findings should be interpreted cautiously, and prospective studies are needed to confirm the benefit and optimal indications for prophylactic EN.

## Highlights

• 35.5% malnutrition rate at 1 month, with mean weight loss of 7.46% at radiotherapy completion.
• Prophylactic enteral nutrition stabilizes weight loss at the end of radiotherapy (*p* < 0.03).
• 31.5% grade ≥ 3 mucositis, associated with T3/T4 stages and extended target volumes.
• 5-year overall survival of 80.8%, unaffected by nutrition support type or radiotherapy modality (IMRT vs. IMPT).

## 1 Introduction

Nasopharyngeal carcinoma is a rare tumor of the upper aerodigestive tract in France (250–300 cases per year). The sex ratio is 2–3 men for every one affected woman, with a median age at diagnosis of 50 years [1], [2], [3]. In France, the standardized 5-year specific overall survival (OS) rate is estimated at 66% [4].

Compared with Squamous Cell Carcinoma (SCC), Undifferentiated Carcinoma of Nasopharyngeal Type (UCNT) affects a younger population and is associated with a more favorable prognosis in terms of local control, progression-free survival (PFS), and overall survival (OS) [5], [6], [7], [8].

The standard treatment for non-metastatic disease includes radiotherapy, delivered with photons (IMRT) or protons (IMPT), either alone or combined with systemic therapy [9], [10], [11].

For high-risk tumors (stage II and III), concomitant chemoradiotherapy (CRT) with cisplatin improves overall survival, with hematologic and gastrointestinal toxicities (mucositis, chemotherapy-induced nausea and vomiting (CINV)) and a requirement for high-grade antiemetic prophylaxis [12]. Carboplatin, used in cases of cisplatin contraindication, has a more limited level of evidence and lower efficacy [13], [14], [15], [16]. Induction chemotherapy, most commonly with TPF (docetaxel, cisplatin, 5-FU) or cisplatin and gemcitabine in the case of UCNT, is recommended for stage III-IV disease with nodal involvement [13], [17], [18].

The nasopharynx is an area at the crossroads of several anatomical structures. Its radiotherapy, alone or combined with concomitant chemotherapy, is associated with toxicities: radiation dermatitis (up to 100% all grades combined), mucositis (up to >30% grade ≥ 3), radiation- and/or chemotherapy-induced nausea and vomiting (RINV and/or CINV, up to 16% grade ≥ 3), esophagitis (up to >25% grade ≥ 2) with functional consequences (xerostomia, hyposalivation or asialia, dysgeusia or ageusia, anosmia, loss of appetite…) [12], [19], [20]. The pathophysiology of RINV, which remains only partially elucidated, likely involves direct interactions between ionizing radiation and chemoreceptors within the dorsal vagal complex (DVC) - including the *area postrema* - as well as the vestibular system (VT), ultimately activating the central vomiting center. With IMRT, these structures, previously minimally exposed to radiation, are now more so, leading to new toxicities [20], [21], [22]. Their dosimetry constraints have not yet been validated in routine practice within guidelines [23], [24], [25], [26].

These toxicities may lead to involuntary weight loss (WL) and the development of malnutrition, two criteria of poor tolerance and poor prognosis in the treatment of nasopharyngeal cancers, up to a 14% difference in OS for a WL cut-off of 5.4% during radiotherapy [19], [27], [28]. Nutritional management is adapted to the toxicities experienced by patients and to their energy and protein requirements, sometimes requiring the prescription of oral nutritional supplements, either the use of enteral nutrition (EN) via nasogastric tube (NGT) or gastrostomy, and/or parenteral nutrition (PN). In oncology, the European Society for Clinical Nutrition and Metabolism (ESPEN) recommends initiating nutritional therapy when oral intake is ≤50% of requirements for more than one week or 50–75% for more than two weeks. In cases of malnutrition with insufficient intake, nutritional support is recommended by the French Society of Clinical Nutrition and Metabolism (SFNCM). [29].

EN via NGT generally represents a temporary option and is initially considered for a short period (<4 weeks), because of discomfort and toxicities, particularly in areas exposed to radiation [30]. PN is indicated in cases of failure or intolerance of EN, or in the presence of severe toxicities, particularly digestive [31].

Some have suggested considering prophylactic gastrostomy EN in the case of diseases and treatments at risk (hypopharyngeal tumor extension, T4 tumor, female sex, CRT…) [31], [32].

Asian teams have demonstrated the benefit of prophylactic EN in nasopharyngeal cancer, making it possible to reduce severe toxicities (grade ≥ 3 mucositis), treatment interruption and extension, and unplanned hospitalizations [33].

The primary objective of the DRAKAR study is to evaluate the benefit of prophylactic EN on the weight change (WC) during, at the end and after radiotherapy for nasopharyngeal cancer from a non-endemic French population. Secondary objectives include assessing the nutritional impact and gastrointestinal toxicities, identification of predictive factors for these toxicities, as well as evaluation of their impact on treatment duration, hospitalizations, and OS.

## 2 Materials and methods

### 2.1 Study design and patients

The DRAKAR study is a retrospective, multicenter, single-arm study conducted in five treatment centers in northwestern France (Cancer Centers of Caen, Lille, Rennes, Rouen, as well as Amiens University Hospital).

Patients received radiotherapy, with or without concurrent chemotherapy, for a primary nasopharyngeal cancer between January 1, 2015, and December 31, 2024. Inclusion criteria were the presence of a primary malignant nasopharyngeal tumor treated with curative-intent radiotherapy (with the tumor in place or postoperatively) using photons or protons, absence of patient opposition to the reuse of medical data for oncology research purposes, and legal adulthood at the time of diagnosis. Non-inclusion criteria were sino-nasal tumors other than nasopharyngeal, hematologic malignancies, sarcomatous tumors, palliative radiotherapy, or treatment of a secondary lesion.

Data were collected retrospectively in the participating centers. Data capture was recorded using the RedCap® electronic case report form software.

### 2.2 Endpoints

The primary endpoint was the percentage change in body weight at the end of radiotherapy (last day of radiotherapy, T2), according to nutritional support status. The percentage of weight change (WC) was also assessed at one month after the start of radiotherapy (T1, D30) and at three months after completion of radiotherapy (T3).

Pretreatment weight loss (WL) was assessed by comparing the usual weight retrospectively recorded in the medical records with the weight measured at the start of radiotherapy (T0: D0). Usual weight generally corresponded to the patient's weight before disease onset, approximately 6 months before diagnosis. Given the retrospective design, the intentionality of WL could not be determined. Weight loss during induction chemotherapy was included in the pretreatment WL calculation.

Malnutrition was defined by the combination of a progressive malignant disease, present in all patients with cancer, and a phenotypic criterion (selected among several provided in the definition), namely WL of 5% in 1 month (or 10% relative to pre-illness weight) for moderate malnutrition, and 10% in 1 month (or 15% relative to pre-illness weight) for severe malnutrition [34]. D30 was a prespecified time point for the secondary assessment of WL and malnutrition, corresponding to the first month of treatment, from day 0 (D0) to day 30 (D30) of radiotherapy.

Secondary endpoints included the rate of moderate or severe malnutrition, pretreatment WL, the rate of gastrointestinal toxicities (grade ≥ 3 mucositis and grade ≥ 3 RINV), the proportion of patients requiring nutritional support (EN, PN), whether prophylactic (before D0 of radiotherapy) or non-prophylactic (initiated during radiotherapy), duration of EN and PN, duration of radiotherapy prolongation related to WL, and the frequency of toxicity-related hospitalizations. Baseline patient characteristics and potential predictors or confounding factors, including personal medical history (chronic alcohol exposure), chemotherapy-related data (regimen and toxicities), and technical and dosimetry parameters (fractionation, radiotherapy modality, target volumes, and DVC and VT doses as potential dosimetry determinant of RINV), were collected and analyzed separately from the study endpoints.

Clinical toxicity data were collected according to the grades of the Common Terminology Criteria for Adverse Events (CTCAE) v5.0.

Dose-volume histograms were generated to extract the delivered doses (minimum, maximum, and mean) to the DVC, as well as the mean and maximum doses and the V40 Gy for the VT and VT + 3 mm [20], [35].

### 2.3 Statistical analysis

Statistical analyses were performed using R software, version 4.4.2. Quantitative variables were described using the mean and standard deviation or the median and quartiles, according to their distribution. Qualitative variables were described using counts and percentages. Comparisons of qualitative variables were performed using the Chi-square test or Fisher's exac*t-*test, and comparisons of quantitative variables using the Student's t-test or the Wilcoxon-Mann-Whitney test, depending on the normality of their distribution. Patient characteristics were described and compared according to the occurrence of nutritional and gastrointestinal toxicities using the statistical tests cited above. Survival curves were estimated using the Kaplan-Meier method and compared using the log-rank test. A *p*-value <0.05 was considered statistically significant.

## 3 Results

### 3.1 Patient characteristics

Among the 183 prescreened patients using the International Classification of Diseases codes C11.0 to C11.9, 124 were included; of the 59 excluded patients, 27 had other histological types (hematological, sarcomatous, or others), 8 were minors, and 24 were treated in a palliative setting and/or underwent re-irradiation.

The cohort was predominantly male (75%), with a mean age of 53 years. UCNT accounted for 59.7% of cases and SCC for 40.3%. Advanced tumor stages (T3 and T4) were frequent (51.6%), as well as nodal involvement (79% of the cases). Fifty-three patients (42.7%) had at least one feature of advanced disease on MRI (meningeal and/or cerebral involvement, midline crossing, or both). SCC showed greater local aggressiveness (advanced T stages, radiological criteria of poor prognosis) than UCNT. Two oligometastatic patients were treated with curative intent. No patient underwent surgical management before radiotherapy **(**Table 1**)**.

**Table 1:** Patient characteristics.

Characteristics	Total N (%), *N* = 124	SCC N (%), *N* = 50	UCNT N (%), *N* = 74	*P-value*
**Sex**				1
Female	31 (25)	13 (26)	18 (24.3)	
Male	93 (75)	37 (74)	56 (75.7)	
**Mean age (years)**	52.6	57.1	49.5	0.001*
**T stage**				0.011*
T1	28 (22.6)	6 (12)	22 (29.7)	
T2	32 (25.8)	10 (20)	22 (29.7)	
T3	23 (18.5)	10 (20)	13 (17.6)	
T4	41 (33.1)	24 (48)	17 (23)	
**N stage**				0.09
N0	26 (21.0)	12 (24)	14 (18.9)	
N1	30 (24.2)	16 (32)	14 (18.9)	
N2	54 (43.5)	20 (40)	34 (45.9)	
N3	14 (11.3)	2 (4)	12 (16.2)	
**M stage**				1
M0	122 (98.4)	49 (98)	73 (98.6)	
M1	2 (1.6)	1 (2)	1 (1.4)	
**Stages**				0.215
Stage IA + IB	24 (19.4)	7 (14)	17 (23)	
Stage II	46 (37.1)	17 (34)	29 (39.2)	
Stage III	52 (41.9)	25 (50)	27 (36.5)	
Stage IV	2 (1.6)	1 (2)	1 (1.4)	
**Tumor in place**	124 (100)			
**Severity criteria**	53 (42.7)	31 (62)	22 (29.7)	0.01*
Meningeal and/or cerebral involvement	13 (10.5)	8 (16)	5 (6.8)	
Crossing of the midline	14 (11.3)	9 (18)	5 (6.8)	
Both	26 (21)	14 (28)	12 (16.2)	
**No severity criteria**	71 (57.3)	19 (38)	52 (71.3)	
Not available	9 (7.3)	3 (6)	6 (8.1)	
No crossing or invasion	62 (50)	16 (32)	46 (62.2)	
**Chronic alcohol exposure**				0.35
Yes	19 (15.3)	10 (20)	9 (12.2)	
No	105 (84.7)	40 (80)	65 (87.8)	

More than half of the population (52.4%) received induction chemotherapy, mainly cisplatin-based (51.2%). The majority of patients (84.7%) were treated with IMRT. Fifteen patients (12.1%) received radiotherapy alone; the remaining patients underwent CRT according to various protocols **(**Table 2**)**.

**Table 2:** Treatment characteristics.

Characteristics	Total, N (%) N = 124
**Radiotherapy modality**	
IMRT (photons)	105 (84.7)
IMPT (protons)	19 (15.3)
**Radiotherapy alone**	15 (12.1)
**Induction chemotherapy**	
Carboplatin-based	2 (1.6)
Cisplatin-based	63 (50.8)
No	59 (47.6)
Grade ≥ 3 CINV	10 (15.9)
Stages I and II (*N* = 70)	31 (44)
Stage III (*N* = 52)	32 (61.5)
Stage IV (*N* = 2)	2 (100)
**CRT regimen**	
Cisplatin-based ≥75 mg/m^2^ per dose	78 (62.9)
Cisplatin-based <74 mg/m^2^ per dose	21 (16.9)
Carboplatin-based	5 (4)
Other	2 (1.6)

### 3.2 Nutritional and gastrointestinal impact

Fifty-nine patients (47.6%) had experienced pretreatment WL compared with their usual weight, including seven (5.6%) with severe malnutrition and three (2.4%) with moderate malnutrition. The mean pretreatment WC was 1.79%, with no significant difference according to whether induction chemotherapy was administered or not (1.66% with and 1.91% without induction chemotherapy, respectively, *p* = 0.84).

At one month (D30) of radiotherapy, 38 patients (30.7%) presented with moderate malnutrition and 6 patients (4.8%) with severe malnutrition, corresponding to an overall malnutrition rate of 35.5%. The mean of WC was −3.79% at D30, −6.45% at the end of radiotherapy, and − 7.79% at three months after completion of treatment. Malnourished patients were significantly younger (48.9 years vs. 54.4 years, *p* < 0.03), with a positive cut-off of 55 years (46% of patients aged <55 years vs. 25% of patients aged ≥55 years, *p* < 0.03). There was no difference in WC either at one month according to radiotherapy modality (mean WC of −3.77% in patients treated with IMRT and − 4.09% with IMPT, *p* = 0.77), or at three months (mean WC of −8.10% with IMRT and − 5.54% with IMPT, *p* = 0.24).

Sixty-nine patients (55.6%) received EN, including 20 (16.1%) prophylactically (18 patients (90%) via gastrostomy and 2 patients (10%) via NGT). EN was mainly delivered via NGT (65.2%), with the remainder via gastrostomy (34.8%). Ten patients (8.1%) received PN, including four (3.2%) after failure of EN. There was no difference in the rate of EN use according to radiotherapy modality (58.5% with IMRT and 38.9% with IMPT, *p* = 0.2). In contrast, the rate of PN use was higher in the IMPT group (44.4%) than in the IMRT group (1.9%, *p* < 0.01). Prophylactic EN and the use of PN allowed stabilization of WL between the end of radiotherapy and three months after completion of radiotherapy. In the absence of prophylactic EN or nutritional support, WL worsened **(**Fig. 1**)**.

**Fig. 1:**
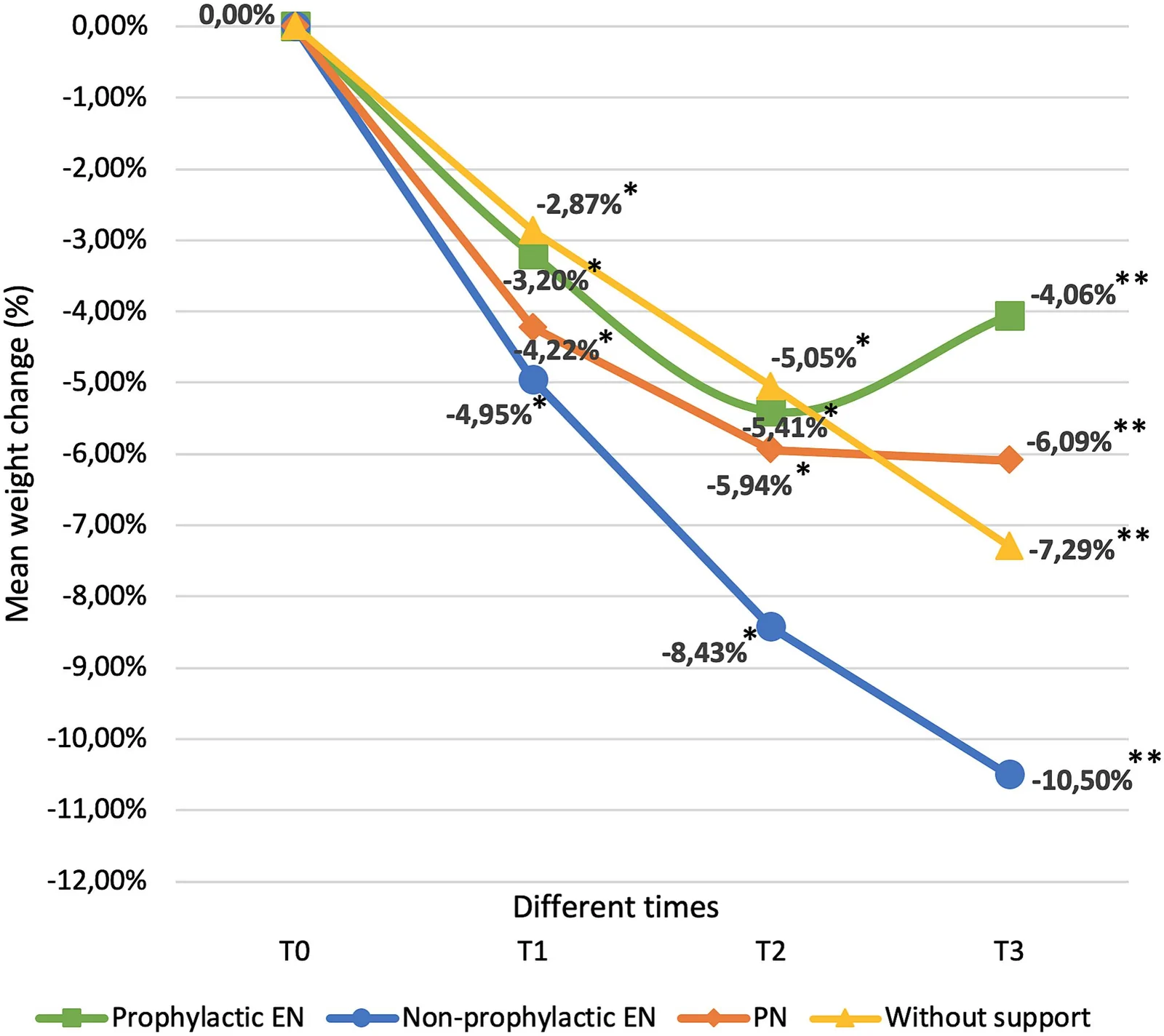
Mean WC according to prophylactic exclusive EN (before D0 of radiotherapy) or non-prophylactic EN, PN (exclusive or after EN failure), or complete absence of nutritional support. (T0: D0 of radiotherapy; T1: at D30 of radiotherapy; T2: at the end of radiotherapy; T3: at three months after completion of radiotherapy); *: *p* < 0.05, **: *p* < 0.01.

Outside the prophylactic EN group, the majority of patients had not experienced pretreatment WL. In the non-prophylactic EN group, only 4 patients (8.7%) did not experience WL between D0 and D30 of radiotherapy **(**Fig. 2**)**.

**Fig. 2:**
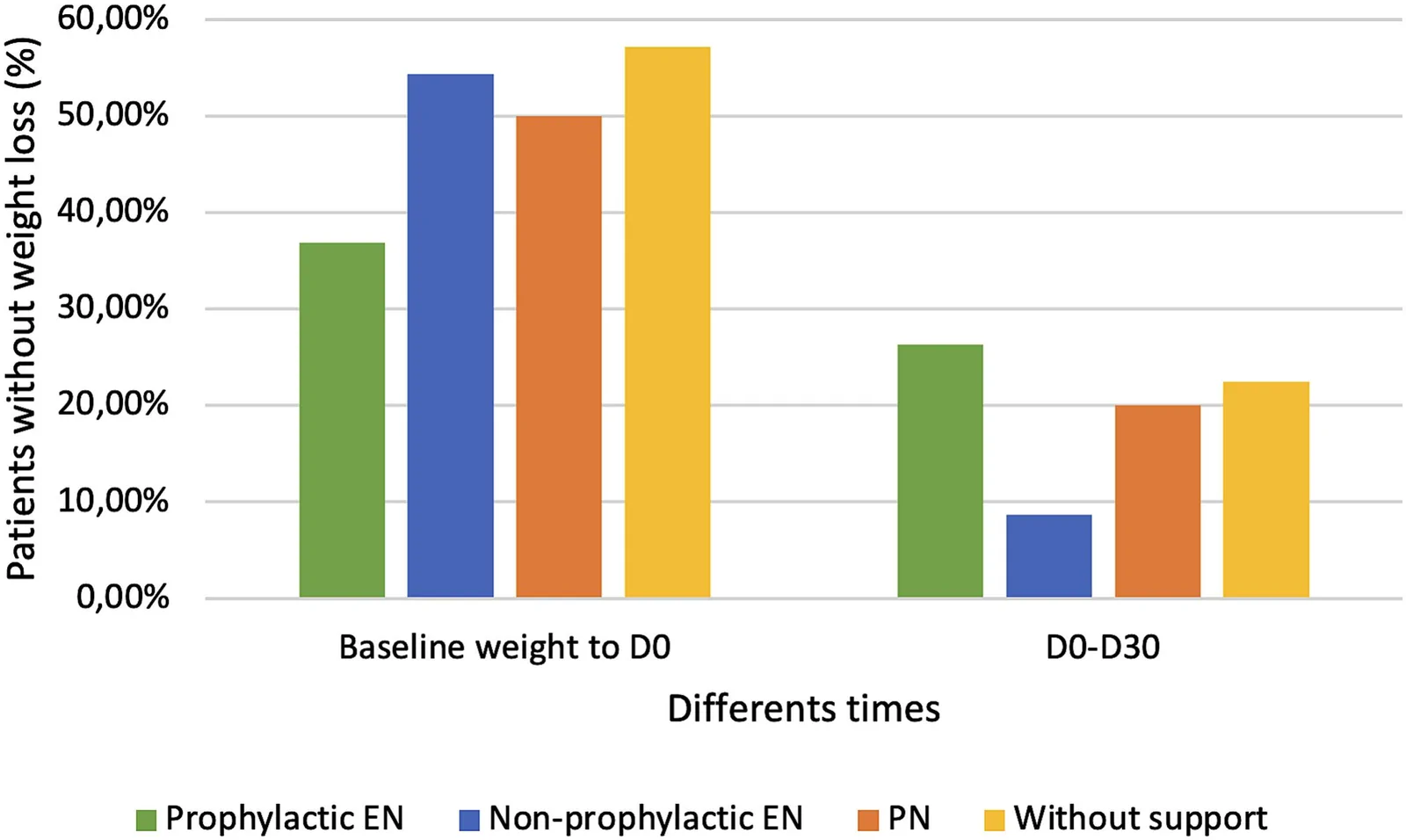
Percentage of patients without WL between usual weight and the start of radiotherapy (usual weight-D0), and between D0 and D30 of radiotherapy (D0-D30).

The prophylactic EN group also exhibited the worse pretreatment WC, with −4.60%, compared with −1.19% in patients without nutritional support, −0.62% in the PN group, and − 1.53% in the non-prophylactic EN group.

The mean duration of EN was 98.9 days (80.1 days for NGT, 148 days for gastrostomy, *p* < 0.01), and 113 days for PN. It was significantly longer with prophylactic EN (mean of 176 days when prophylactic versus 72.5 days when non-prophylactic, *p* < 0.01).

For the 28 patients analyzed, doses to the DVC were significantly lower in the IMPT group, with a mean dose of 17.7 Gy versus 29.4 Gy with IMRT (*p* < 0.01), a mean maximum dose of 20.9 Gy versus 31 Gy (*p* < 0.02), and a mean minimum dose of 15.1 Gy versus 28.2 Gy (*p* < 0.001). Maximum doses, mean doses, and the V40 Gy percentage of the VT and VT + 3 mm were similar in both groups.

The rate of grade ≥ 3 mucositis was 31.5% (*N* = 39) and was associated with intermediate-risk CTV volume (*p* < 0.01) and T3–T4 stage (*p* < 0.01).

The rate of grade ≥ 3 RINV was 14.5% (*N* = 18); female sex was significantly associated (*p* < 0.01). No association was found with dosimetric parameters, including target volumes, doses to the DVC or VT, or inclusion of the cavernous sinus or clivus, nor with chronic alcohol exposure.

Radiotherapy alone was significantly less toxic in the pooled analysis of digestive toxicities (mucositis and grade ≥ 3 RINV), with 13.3% versus 43.1% (*p* = 0.04).

In univariate analysis, no significant association was found between digestive and nutritional toxicities (defined by malnutrition) and: age (cut-off at 55 years), histology, maximum grade of CINV during induction chemotherapy, dosimetric criteria, type of adjuvant chemotherapy regimen, or radiotherapy modality (IMRT or IMPT).

In multivariate analysis, age < 55 years was independently associated with a higher risk of moderate or severe malnutrition at 1 month (OR 2.62, 95% CI 1.21–5.88; *p* = 0.016), after adjustment for sex and prophylactic nutritional support. A non-significant trend toward a lower risk was observed for male sex and prophylactic nutritional support, whereas pretreatment WL showed a non-significant trend toward higher odds of malnutrition (Table 3).

**Table 3:** Multivariate analysis of factors associated with moderate or severe malnutrition at 1 month of radiotherapy. OR, odds ratio; CI, confidence interval. Reference categories were female sex, age ≥ 55 years, absence of prophylactic nutritional support and no pretreatment WL.

Characteristics	OR	95% CI	p
**Patient sex**			
Women	–	–	–
Male	0.51	0.21, 1.23	0.14
**Age**			
≥55 years	–	–	–
<55 years	2.62	1.21, 5.88	0.016
**Prophylactic nutritional support**			
No	–	–	–
Yes	0.50	0.15, 1.42	0.2
**Pretreatment weight loss**			
No	–	–	–
Yes	1.54	0.70, 3.41	0.3

No significant interaction was observed between prophylactic nutritional support and pretreatment WL (*p* = 0.90), indicating that the association between prophylactic nutritional support and malnutrition at 1 month did not differ according to the presence or absence of pretreatment WL.

### 3.3 Treatment course

Three patients underwent prolonged treatment due to interruption (4 and 6 days) related to toxicity. No association was found between these interruptions and nutritional or digestive toxicity (*p* = 0.56 and 0.57).

Thirty patients (24.2%) were hospitalized during radiotherapy, mainly those receiving nutritional support (32.3% of patients with EN, 80% of patients with PN, 2% of patients without support, *p* < 0.001). Prophylactic EN was not protective against hospitalization, with 25% of hospitalized patients having prophylactic EN versus 75% non-prophylactic EN, with no significant difference (*p* = 0.79).

### 3.4 Survival data

Three-year OS was 86% (94% for UCNT, 73% for SCC), and five-year OS was 80.8% (88% for UCNT, 69% for SCC), with no difference according to radiotherapy modality (*p* = 0.089) **(**Fig. 3**)**.

**Fig. 3:**
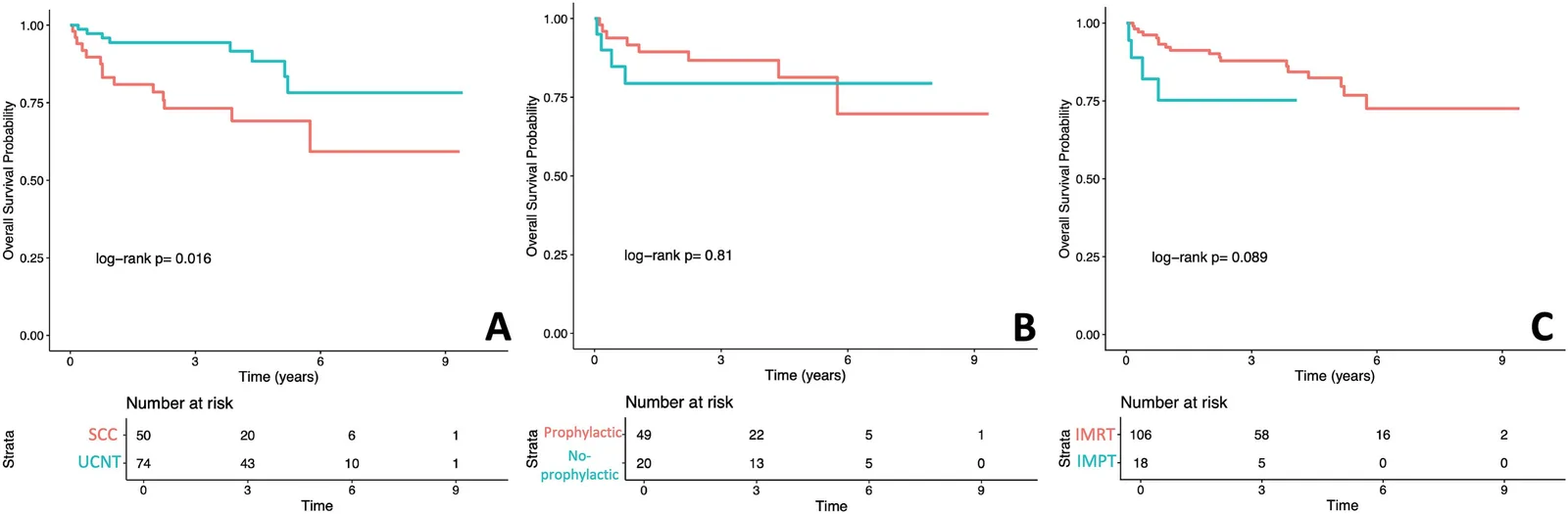
Survival curves according to histological subtype (SCC versus UCNT) (A), prophylactic EN or not (B), and radiotherapy modality (IMRT versus IMPT) (C).

Patients who received EN showed no difference in OS, according to whether it was prophylactic or not (79.4% at 5 years versus 81.3%, respectively; *p* = 0.81).

Patients treated with IMRT showed no difference in OS compared with those treated with IMPT (87.9% at 3 years versus 75.2%, respectively; *p* = 0.18).

## 4 Discussion

This multicenter French study provides contemporary real-world data on the nutritional and digestive burden of radiotherapy for nasopharyngeal cancer in a non-endemic setting. More than one-third of the patients experienced clinically relevant malnutrition within the first month of treatment, confirming the substantial supportive care needs in this population.

Our findings suggest that prophylactic EN may contribute to limiting post-treatment WC, particularly in patients identified as nutritionally vulnerable at baseline. The high nutritional and digestive impact observed in this study was reflected by a high rate of moderate and severe malnutrition, a high frequency of hospitalizations, and a high incidence of grade ≥ 3 toxicities. Apart from younger age, with a cut-off at 55 years, no predictive factor for malnutrition was identified in this series. In accordance with the literature, WC during radiotherapy was substantial and comparable to that reported in an Asian patient population (−6.85% versus −6.45% in DRAKAR) [19]. Similarly, female sex was associated with a higher incidence of RINV.

Supportive care allowed mitigation of the high rates of malnutrition and grade ≥ 3 toxicities, without compromising treatment continuity or completion of the planned treatment schedule.

The difference in 5-year OS between UCNT and SCC (88% vs. 69%) was consistent with the previously reported more favorable prognosis of UCNT and was not further explored in this study.

Oropharyngeal cancers share anatomical features and treatment modalities with nasopharyngeal cancers, leading to similar digestive and nutritional toxicity profiles [36].

A Swiss prospective study of 45 patients with oropharyngeal cancer treated with radiotherapy alone showed that prophylactic EN via gastrostomy, based on pretreatment severity criteria (WL ≥10% of usual body weight, BMI <20 kg/m^2^, age ≥ 70 years), reduced hospitalizations for dehydration and WL by approximately 3%, with no available survival data [37]. Among patients receiving non-prophylactic EN, 54.3% had no pretreatment WL but exhibited the greatest final WL, suggesting that non-prophylactic EN may not compensate for established nutritional toxicity. Conversely, prophylactic EN was used in patients with the greatest pretreatment WL and marked nutritional vulnerability (63.2% had experienced WL before treatment). These findings are further informed by a retrospective Thai study of 904 patients with nasopharyngeal cancer, which found no difference in WL at the end of CRT according to prophylactic EN use [38]. However, BMI <18.5 kg/m^2^ was independently associated with greater benefit from prophylactic EN (coefficient 4.16, 95% CI 1.08–7.24), a finding not observed in our cohort. These findings suggest that absence of pretreatment WL does not reliably identify patients at low risk of nutritional toxicity. Finally, a retrospective Chinese study of patients with nasopharyngeal cancer reported a benefit of prophylactic EN in terms of OS and PFS among patients with N3 disease, without a significant difference in the overall cohort of 133 patients [39]. In our cohort, better post-treatment weight recovery in the prophylactic EN group supports its potential role in nasopharyngeal radiotherapy, particularly in patients potentially vulnerable from a nutritional and digestive standpoint: younger age, CRT, female sex (pretreatment WL, pre-existing malnutrition, or N3 stage were reported in the literature but were not significant in our cohort). Regarding PN use, the higher rate in the IMPT group (42.1% vs. 1.9% with IMRT) likely reflects center-specific nutritional or technical practices, as IMPT was performed at the only protontherapy center among the five participating centers.

Caution is warranted when deciding whether to use a gastrostomy rather than an NGT. In head and neck cancers, the available literature appears to support broadly comparable outcomes between the two techniques [40]. Gastrostomy may offer advantages in terms of patient tolerance and potentially quality of life, although this remains debated in head and neck cancers [41], [42]. However, gastrostomy is also associated with a longer duration of EN use, particularly when used prophylactically, as observed in our cohort, which may affect patient burden and quality of life. In NPC, data remain limited; notably, a Chinese cross-sectional study of 148 patients found no significant difference in long-term quality of life according to gastrostomy use after chemoradiotherapy [43]. Our study was not designed to assess these outcomes.

Absence of a significant increase in treatment duration related to toxicities was also reported in a French series of oropharyngeal cancer treatment [44].

Regarding the relationship between irradiated volume size and the severity of observed toxicities, in our study, only the intermediate risk CTV volume emerged as a significant factor, likely due to its greater variability according to disease stage compared with other volumes. The results of the phase III trial conducted by *Xiang* et al. are consistent with this finding: a reduction in irradiated volumes using the post-induction chemotherapy GTV as GTV-T resulted in lower rates of mucositis, reduced use of EN, and less WL during and at the end of radiotherapy compared with a GTV based on pre-chemotherapy volumes [45].

The absence of a significant association between dosimetry parameters and severe gastrointestinal toxicities may reflect limited statistical power and the focus on grade ≥ 3 events, which was deliberately chosen to limit misclassification related to retrospective toxicity ascertainment. Indeed, *Lee* et al. reported, for nasopharyngeal radiotherapy, an association with grade I–II RINV for a V40Gy ≥80%, without any grade 3 toxicity [20]. In contrast, dose–effect relationships have been reported for dysphagia in nasopharyngeal cancer, with radiation dose to the pharyngeal constrictors associated with swallowing impairment [46]. Similarly, the dosimetry benefit of IMPT was observed in our study, without clinical translation in terms of grade ≥ 3 RINV. This may be related to a limited sample size (19 patients in the IMPT group and 11 analyzed in the IMRT group), or to a lack of relevance of DVC dose as a predictor of RINV in this indication. The hypothesis of a referral bias toward IMPT in patients with advanced-stage disease was not confirmed in our study (47.6% of T3-T4 stages in IMRT versus 73.7% in IMPT, *p* = 0.18); however, IMPT indication is primarily based on the involved anatomical areas rather than the stage alone.

Our study has several limitations, notably its retrospective design, which may have led to missing data and heterogeneity in the collection of toxicity and weight data during treatment, as well as a limited sample size. Patients receiving prophylactic EN had greater pretreatment WL, and therefore treatment causality cannot be inferred. In addition, practices evolved across centers over the ten-year study period. Adherence to ESPEN/SFNCM criteria could not be verified retrospectively, and local practice variability between centers cannot be excluded. Although WL is a diagnostic criterion for malnutrition according to the HAS criteria, its use alone to assess overall nutritional status is insufficient; it would have been of interest to include other criteria, such as sarcopenia assessed by bioelectrical impedance analysis, or physical strength evaluation using handgrip testing or gait speed test, as well as biological follow-up data (serum albumin and protein levels) [47]. Finally, nutritional management is combined with recommendations for adapted physical activity and dysphagia rehabilitation in the relevant patients, extending throughout the entire course of care [31], [48]. These results should be interpreted as hypothesis-generating. Future perspectives of this research could include a prospective evaluation incorporating long-term weight parameters, as well as measures of physical strength and an assessment of patient quality of life.

## 5 Conclusion

In patients undergoing radiotherapy for nasopharyngeal cancer, substantial weight loss and acute digestive toxicities remain frequent despite modern treatment techniques. In this retrospective multicenter cohort, prophylactic enteral nutrition was associated with post-treatment weight stabilization but did not reduce hospitalization rates or improve overall survival.

These findings suggest that early nutritional support may benefit selected high-risk patients (younger age, CRT, female sex), but the greater pretreatment weight loss observed among patients receiving prophylactic enteral nutrition indicates potential confounding by indication and limits causal interpretation. Prospective studies incorporating functional and quality-of-life outcomes are required to establish optimal supportive care strategies.

## CRediT authorship contribution statement

**Thomas Cochin:** Writing – review & editing, Writing – original draft, Methodology, Conceptualization. **Jennifer Le Guévelou:** Writing – review & editing. **Juliette Thariat:** Writing – review & editing. **Justine Lequesne:** Writing – review & editing, Writing – original draft, Formal analysis. **Sébastien Thureau:** Writing – review & editing. **Alexandre Coutte:** Writing – review & editing. **Jacques Balosso:** Writing – review & editing. **Jonathan Lichtlé:** Writing – review & editing. **Emmanuel Kammerer:** Writing – review & editing. **Joël Castelli:** Writing – review & editing. **Xavier Liem:** Writing – review & editing. **Sylvie Khan:** Writing – review & editing. **Audrey Rambeau:** Writing – review & editing, Writing – original draft, Conceptualization. **Audrey Larnaudie:** Writing – review & editing, Writing – original draft, Conceptualization.

## Ethical approval

Based on the submitted documents, the Scientific and Ethics Committee of the François Baclesse Center issued a favorable opinion for this study on June 19, 2024. In accordance with current regulations, this study was declared compliant with Reference Methodology MR-004 by the French Data Protection Authority (Commission Nationale de l'Informatique et des Libertés; declaration number: 2214228v0).

## Ethics declaration

### Informed consent and patient details

Written informed consent to take part in the study and to publish the article has been obtained from all participants or their legal representatives. The privacy rights of participants have been observed.

### Studies in Human

This study was performed in compliance with relevant laws, regulatory frameworks and guidelines where the research took place. This study was approved by the Comité Scientifique et Ethique François Baclesse and Commission Nationale Informatique et Liberté. (Approval No. 2214228v0)

## Funding

The authors declare that no funding was received for this work.

## Declaration of competing interest

The authors declare that they have no known competing financial interests or personal relationships that could have appeared to influence the work reported in this paper.
